# Do Degrees Matter? Rethinking Workforce Development for Youth with Intellectual Disabilities and Mental Health Challenges

**DOI:** 10.1007/s43477-023-00076-5

**Published:** 2023-03-23

**Authors:** Susan Reay, William Reay, Kris Tevis, Lisa Patterson

**Affiliations:** 1 Grace Abbott School of Social Work, University of Nebraska Omaha, 6001 Dodge St., CPACS 205, Omaha, NE 68182 USA; 2 Omni Inventive Care, Omaha, NE USA

**Keywords:** Training, Mental health, Intellectual disability, Education, Task shifting

## Abstract

The global workforce crisis significantly impacts how evidence-based treatment is provided to youth with developmental disabilities and co-occurring mental health conditions. Addressing the workforce crisis requires re-examining the long-standing methods of selecting individuals for employment based on academic degrees. This project offers an innovative workforce development option that provides specialized training to staff with advanced education degrees and staff with less education. The participants in this study were employed in a rural area of the USA within the mental health, child welfare, and correctional industries. All participants worked with youth experiencing intellectual disabilities and mental illness. Results indicated that participants improved their knowledge of the population, demonstrated a better understanding of EBPs, and were willing to employ evidence-based approaches regardless of their education or age. Although overall attitudes toward EBPs decreased, diverging attitudes increased, suggesting a need to accommodate treatment strategies when EBP models are unavailable for special populations. Initial knowledge gaps demonstrated by those with a master's degree and those with less education disappeared after the training. This finding supports the application of innovative task-shifting options in mental health, such as diverting more sophisticated care tasks to nonprofessionally trained persons, which can reduce workforce pressure and unmet demand for care. This study demonstrates cost-effective and time-efficient methods of training staff regardless of education by relying less on specific EBP models and more on adaptation.

## Do Degrees Matter?

Youth with intellectual or developmental disabilities (IDD) represent a unique population with an array of physical, mental, and educational needs that consequently impact their care and treatment. In 2013, national estimates for the USA identified 6.2 million people live with an IDD, over 4 million of which are children (Larson et al., 2016), which equates to almost 7% of all children are diagnosed with IDD (Zablotsky et al., 2015).

Higher rates of mental and physical illnesses are associated with youth and adults with IDD (World Bank, 2022). In this population, comorbidity occurs at an earlier age than the general public and includes a range of mental and physical conditions with different levels of impairment (Cooper et al., 2015). Varying greatly, research examining the prevalence of mental illnesses as a co-occurring disorder in people with IDD indicates that 13.2% to 74% of individuals with IDD also have a mental illness (Buckles et al., 2013). The large variability in the research is a result of different sampling methodologies, participant selection criteria, diagnostic classification system, measurement tools, and methods used to define IDD and mental illness (Hemmings & Bouras, 2016).

Compounding the complexity of factors associated with health inequalities and co-morbidities with this population, a long-standing workforce crisis in the USA and across the globe presents challenges to service delivery (Bogenschutz et al., 2014; World Bank, 2022). The need for direct care workforce has nearly doubled in the last decade in the USA, going from 2.9 million workers in 2008 to almost 4.5 million workers in 2022 (Snyder & Espinoza, 2022). The next 10 years is expected to require an additional 1.8 million positions. However, the field is plagued by consistently low wages, particularly for direct care staff (Bogenschutz et al., 2014; Hewitt et al., 2008). The impact of COVID-19 has compounded this phenomena. More people need care than ever before and the care provided can be complex and long-lasting (Johnson, 2022). Salaries in non-healthcare-related fields have risen considerably because of the workforce shortage in all areas of commerce (Johnson, 2022). Thus, making it harder to attract and sustain a healthcare workforce because individuals employed in healthcare have skills that are easily transferred to other work sectors that pay considerably more and have less demanding responsibilities (Hussein & Turnpenny, 2020). Wages for individuals providing care to individuals with intellectual disabilities and mental health challenges are associated with high levels of poverty; therefore, it is not surprising that staff turn-over rates are reported as high as 43.8%. (Friedman, 2021; Ruggles et al., 2019).

Historically, direct service providers have insufficient training and preparation for job duties (President’s Committee for People with Intellectual Disabilities, 2017). Women and individuals of color are overrepresented in the workforce, many of whom experience educational barriers (Campbell, 2017). Most of the workforce have bachelor’s degree or below and thus have less exposure to opportunities to increase knowledge acquisition and skills in working with individuals with complex needs (Friedman, 2021). Yet, some service provision is provided by staff with a master’s degree or higher. Staff with a master’s degree or higher are generally in administration, clinical, or therapeutic roles that supervise direct care staff and coordinate service provision. Typically, master’s degree wages are higher and have more flexible work hours, benefits, and support. Due to their formal education, staff with master’s degrees or higher receive the most training. In addition to their college courses, individuals with master’s degrees generally have increased opportunity for continuing education that has the potential to influence their knowledge, skills, support, and attitude in working with this population.

However, knowledge deficits exist with staff who have master’s degrees as well. Williams and Haranin (2016) found that therapists with master’s degrees lack confidence in diagnosing and treating this population and only half of mental health therapists received any training in IDD/MH in their academic career. Only 16% of staff with master’s degrees working with autistic youth reported receiving supervision from someone with expertise with this population (Williams & Haranin, 2016). This outcome is concerning given that training programs, regardless of the staff’s degree or experience, are highly correlated with a positive attitude toward the consumer population. Positive staff attitudes significantly influence service delivery, staff turn-over rates, and quality of life for consumers and the employee population (President’s Committee for People with Intellectual Disabilities, 2017).

Enhancing staff knowledge has demonstrated improvements in the quality of care they provide to consumers (Whitehurst, 2008). When staffs’ knowledge increases, stigmatizing attitudes about people with IDD decreases (Hampton & Xiao, 2008). Without a doubt, training is an essential part of workforce development. Training also has a positive influence on the staff’s well-being. In addition to enhanced performance, training improves staffs’ psychological well-being and lowers rates of burnout (Chung & Harding, 2009).

The present study explores the potential for mental healthcare task shifting through training offered by an individualized online training platform. This study aimed to determine the effectiveness of a state-wide, virtual educational program in increasing participants’ attitudes and knowledge of evidence-based treatment to care for individuals with IDD and mental health conditions. We hypothesize that participants will achieve higher knowledge scores after completing the training when compared to their pre-training EBP knowledge regardless of their level of formal education prior to the training. Furthermore, we hypothesize that participant’s age, gender, and attitudes toward EBPs would not affect his or her EBP knowledge after the training.

## Methods

### Procedure

In the spring of 2021, Omni Inventive Care collaborated to provide a virtual training titled, “Low Cognitive Disorders and Mental Illness.” The training was provided by a content matter expert, William Reay, PhD. in research and mental health service delivery. The one-day virtual training targeting providers in Nebraska, United States, lasted seven hours with two fifteen-minute breaks and a half hour break for lunch.

Participants attended the training at no cost but were required to register to gain access to the pre-test and log-on information. Pre-tests were completed up to a week prior to the training. Funding for the training was covered by a federal, state, and local partnership that included state funds and a non-profit community-based mental health agency. Participants received continuing education credit across several disciplines.

Participants were instructed to complete a pre-test and post-test, which included an evidence-based attitude scale and an evidence-based practices knowledge test administered virtually through QuestionPro. Demographic information about the participants was also collected at this time. Participants received e-mail reminders to complete the pre-test prior to the training, verbal reminders during the training, and an e-mail after the training to complete the post-test. Post-tests were completed within a week after the training. Institutional review board approval (0458-22EX) was granted through the University of Nebraska at Omaha, United States.

### Participants

Two hundred twelve participants attended the training, with demographic information obtained from 198 participants. Of those who attended, 180 completed the pre-test, while 136 of the initial 180 individuals finished the post-test. One hundred fourteen individuals completed both the pre- and post-test. Females accounted for 80% (*N* = 158) of participants. Eighty-nine percent of attendees reported their race as White (*N* = 177), 6% reported as Black (*N* = 11), 2% reported as Hispanic/Latino (*N* = 3), and 3% reported their race as Asian/Pacific Islander, Native American, or bi-racial (*N* = 7). Although the training did not target any particular licensure or educational level, the majority of participants held a master’s degree or higher (79%) and held at least one mental health practice license (86%). Participants worked in a variety of environments, but the most common included case management, government, criminal justice/probation, social work, outpatient clinics, private practice, outpatient substance abuse, and inpatient psychiatric units. Sixty-one percent of attendees reported being under 51 years old. Tables 1 and 2 summarize the demographic data of training participants.

**Table 1:** Sociodemographic characteristics of participants

Variable	Univariate statistics
Age	
18–30 years	27 (13%)
31–40 years	45 (23%)
41–50 years	49 (25%)
51 years & older	77 (39%)
Sex	
Male	40 (20%)
Female	158 (80%)
Highest education level	
High School graduate/equivalent	1 (1%)
Some college, no degree	4 (2%)
Associate’s degree	9 (4%)
Bachelor’s degree	26 (13%)
Master’s degree	140 (70%)
Professional degree	2 (1%)
Doctorate degree	16 (8%)

*N* = 198

**Table 2:** Participant work environment and licensure

Variable	Univariate statistics
Work Environment	
Agency Outpatient	57 (29%)
Case Manager	34 (17%)
Inpatient Psychiatric	31 (16%)
Criminal Justice/Probation	30 (15%)
Outpatient Substance Abuse	30 (15%)
Other^a^	29 (15%)
Private Practice Outpatient	23 (12%)
Government	18 (9%)
Residential Substance Abuse	11 (5%)
Social Work—Medical	8 (6%)
Social Work—Schools	6 (3%)
Macro^b^	4 (2%)

*N* = 198. Participants may have more than one work environment

^a^Reflects other environments, such as those who are consulting, retired, mental health residential, administration/management, rehabilitation programs, and education/teaching

^b^Reflects individuals who may be working in program evaluation, program development, policy, advocacy, etc

### Measures

Two measures were used in the study which included the Evidence-Based Practice Attitude Scale (EBPAS) and a competency exam designed by the researchers. The EBPAS measures individual’s attitudes toward evidence-based practices. A 15-item instrument, the EBPAS asks participants to respond on a four-point Likert scale (0 = “not at all” to 4 “to a very great extent”) to the extent to which they agree to a particular statement related to evidence-based practices (Aarons, 2004). Higher scores reflect a more favorable attitude. In addition to an overall score, the EBPAS generates four subscales, including (a) appeal—appeal of EBPs, (b) requirements—EBP use is required by the individual’s organization, (c) openness—openness to trying EBPs, and (d) divergence—unfavorable attitudes toward EBPs. Summed scores generate an overall total score ranging from 0 to 60. For cumulative scores, the divergence scale is reverse scored. A nationwide study of mental health service providers showed evidence for good internal consistency on the full scale and (α = 0.82) and all subscales ranging from 0.72 to 0.94 (divergence, α = 0.72; appeal α = 0.76; openness, α = 0.81; requirements, α = 0.94) (Aarons et al., 2010).

EBP knowledge and content competency were evaluated through the use of a knowledge test administered prior to the training and immediately after the training. The researchers designed 23 questions based on the training curriculum and feedback from subject matter experts. Participants earned one point for each correct answer for a total possible score of 23 points (See Appendix for a list of questions).

### Training Description

This daylong presentation employed best practices in adult education, which included the influence of the cognitive psychological scholarship provided by Reason and Hobbs (2017). This training offered the best available evidence-based treatment information, hierarchically arranged to build upon concepts and data. The training referred to in the present study was designed to provide participants with information on EBPs; how to conceptualize cases in terms of manageable adaptations (i.e., strategies adaptable to various client characteristics and circumstances initially excluded from original EBP studies); and workable clinical applications associated with engaging and treating multi-comorbid individuals with both intellectual disabilities and serious mental illness.

The first topic covered was *Common Child and Youth Management Understandings*. The presenter reviewed caregiver management styles along with how individuals respond to elements of caregiving, such as nurturing, engagement, and retribution. Core elements of common parenting styles can apply in staff and client interactions of all ages considering the caregiving nature of the relationship. The intricate balance of demandingness and responsiveness certainly influences an individual’s reaction to basic commands in a variety of environments and roles. However, these delicate interactions set the stage for ongoing relationships and development of therapeutic alliance.

The second topic includes training on *Promising Practices and Practices to Avoid*. The presenter focused on evidence-based practices (EBPs), the active elements of EBPs, and discredited practices. Some questions that were addressed in this topic were, “Why do we want to use Evidence-Based Treatments and promising practices?” and “Are there known harmful practices and treatments that are currently being used?” The topic began with the history of the field of mental health and moved to the current connection between mental health science and practice. Participants learned that EBP is a process of evaluating evidence, not just a manualized treatment, which is a critical distinction when EBPs have not been developed for certain populations, and adaptation and accommodations are required.

The third topic of the training, *Prevalence of Co-Occurring Intellectual/ Developmental Disabilities and Mental Health Disorders and The Process of Engagement* gave context to the pervasiveness of adults and children with IDD and a co-occurring mental health disorder. The presenter reviewed why engagement between the therapist/caregiver and client must be established but also difficult to achieve. Participants learned evidence-based engagement strategies that are effective in working with this population and adaptations of care.

The training concluded with the topics of *The Importance of Using Data and Assessing Behavior and Using Functional Behavior Assessments* because of the importance of collecting and analyzing data to improve outcomes for clients and to highlight the essential functions of clinical self-evaluation (Connors et al., 2021). The presentation offered examples of cost-effective evidence-based assessment tools used in clinical settings for multi-morbid individuals. Lastly, the presenter introduced components of Functional Behavioral Assessments (FBA) to show participants how to identify and treat challenging behaviors, which is prevalent with among individuals with IDD. Participants were provided an opportunity to ask questions with the presenter at the conclusion of the training.

### Data Analysis Methods

The Statistical Package for Social Sciences (SPSS) for windows, version 28 was used to obtain descriptive information about the participants and to test study hypotheses. The characteristics of subjects and assessment variables were analyzed through descriptive statistics to generate a general description of participants. Simple pre- and post-test comparisons were analyzed through paired sample *t* tests. The relationship between EBP knowledge, attitudes, and categorical variables such as highest degree earned, gender, and age were examined through correlations and mixed group factorial ANCOVA. Due to limited variability, categories for participant education were collapsed to capture individuals with a bachelor’s degree or lower and those with a master’s degree or higher. Participant’s age was also collapsed into bivariate categories of those under 50 years old and those 51 years and older.

## Results

Although two hundred twenty-one individuals attended at least a portion of the training, one hundred eighty individuals completed the pre-test and one hundred fourteen participants completed both the pre- and post-training exams. Since the training was offered online, the pre-test and post-test were administered online as well. Despite several reminders some individuals did not complete the competency tests, but 56% offered demographic data which was comparable to those who completed the competency exams. Approximately 56% of non-responders were less than 50 years old and 6% were male and 61% had a master’s degree or higher. Eighty-three percent of the non-responders were White, 11% were Black, and 6% reported a mixed-race background. Almost all of the non-responders (94%) worked in the mental health field. Post-test data were not collected from individuals who did not attend the entire training. Since the training lasted 7 h, some individuals could not commit to the entire day. Therefore, those who participated for only a portion of the training were not included in the pre- and post-test comparisons. Individuals who completed less than 50% of the pre-test were also excluded.

Pre-training exam scores ranged from 4 to 22, with an average score of 11.67 (SD = 3.150). While gaining an average of 3.325 points, post-training scores ranged from 7 to 20 (*M* = 14.99, SD = 2.692). As hypothesized, participants scored higher on the post-training EBP knowledge exam *t*(113) = − 10.017, *p* = 0.000, using paired sample *t* test analysis. Summary statistics for pre- and post-training knowledge for participants are displayed in Table 3.

**Table 3:** Descriptive statistics for pre- and post-training EBP knowledge

Baseline characteristic	Pre-training				Post-training			
	*n*	*M*	SD	Range	*n*	*M*	SD	Range
Gender								
Male	39	11.08	3.132	4–17	28	14.29	2.917	10–20
Female	141	11.72	3.269	5–22	92	15.15	2.572	7–20
Age								
50 years or less	111	11.27	3.060	5–19	71	15.24	2.399	10–20
51 years of more	69	12.09	3.480	4–22	54	14.04	2.835	7–19
Level of education								
Bachelor’s degree or less	33	9.58	2.969	4–17	23	15.00	2.876	9–19
Master’s degree or more	147	12.03	3.137	5–22	102	14.66	2.612	7–20

*N* = 180 for pre-training; *N* = 125 for post-training

Although still lacking uniformity, some human services, counseling, social work, and psychology programs have begun introducing EBPs into their educational curriculum (Substance Abuse & Mental Health Service Association, 2022). At the very least, those finishing graduate educational courses recently have at least minimal exposure to EBP programs or interventions by name. Consistent with this assumption, a 2 × 2 mixed groups factorial was performed to examine the effects of level of education (master’s degree or higher, bachelor’s degree or lower) and testing (pre-test, post-test) competency. Table 4 shows the pre- and post-test means by education level.

**Table 4:** Pre- and post-test means by education level

Testing	Level of education	
	Master’s or higher (*n* = 93)	Bachelor’s or lower (*n* = 16)
Pre-test	11.95	10.12
Post-test	14.71	15.63

*N* = 109

Results show an interaction between level of education and testing (*F*(1, 107) = 5.779, *p* = 0.018, *Mse* = 8.845). Further analysis based on LSD follow-ups of the cell means (minimum mean difference = 1.627) revealed that the pattern of this interaction shows individuals consistently scored lower on pre-test performance and higher on post-test performance as hypothesized. Although individuals with more education, those with at least a master’s degree, scored higher on the pre-test than individuals with less education, no difference in scores appeared on post-test performance.

There was a significant main effect for time of testing (*F*(1, 107) = 52.696, *p* < 0.001), with better overall performance on the post-test competency test as hypothesized. This main effect is descriptive for both age groups (LSD minimum mean difference = 1.140). There was a nonsignificant main effect for level of education (*F*(1, 107) = 0.784, *p* = 0.378) showing no significant difference between overall performance between the educational levels. However, the main effect is descriptive only for the post-test and misleading when compared to the pre-test results (LSD minimum mean difference = 1.025). When comparing simple effects, individuals with high levels of education scored similarly to participants with less education on the post-test, but significantly higher on pre-test performance.

Many factors contribute to how information is absorbed. To further examine the relationship with level of education a 2 × 2 mixed groups factorial was performed with level of education (master’s degree or higher, bachelor’s degree or lower) and testing (pre-test, post-test) competency while controlling for the effects of age and gender.

Results show a marginally significant interaction between level of education and testing (*F*(1, 105) = 3.489, *p* = 0.065, *Mse* = 8.393). Further analysis based on LSD follow-ups of the cell means (minimum mean difference = 1.578) revealed the pattern of this interaction shows individuals consistently scored lower on pre-test performance and higher on post-test performance as hypothesized. Although individuals with more education and those with at least a master’s degree scored higher on the pre-test than individuals with less education, no difference in scores appeared on post-test performance.

Consistent with previous results, there was a significant main effect for time of testing (*F*(1, 105) = 12.219, *p* < 0.001), with better overall performance on the post-test competency test as hypothesized. This main effect is descriptive for both age groups (LSD minimum mean difference = 1.116). There was a nonsignificant main effect for level of education (*F*(1, 105) = 1.069, *p* = 0.304) showing no significant difference between overall performance and the educational levels. However, the main effect is descriptive only for the post-test and misleading when compared to the pre-test results (LSD minimum mean difference = 1.0357). When comparing simple effects, individuals with high levels of education scored similarly to participants with less education on the post-test, but significantly higher on pre-test performance. The results of this study support the notion that clinicians with a graduate education have more knowledge of EBPs than those with less education. However, these differences vanish after a one-day training program. As hypothesized, the training improved knowledge of EBPs regardless of the participant’s level of formal education at the time of the training but enhanced knowledge more for individuals with no graduate training while controlling for age and gender.

Lastly, attitudes clearly influence behavior, so the present study explored EBP knowledge gained through virtual training and its relationship with existing attitudes toward EBPs as prior knowledge may bias consumption of information. As hypothesized, correlational analysis revealed no significant relationship with post-training EBP knowledge and overall attitudes toward EBPs or any of the EBPAS subscales. However, participant’s attitudes toward EBPs changed after the training. Using paired sample t test analyses, participant’s overall attitudes (*t*(115) = 15.26, *p* < 0.001) as well as attitudes about EBP requirements (*t*(115) = 3.34, *p* = 0.001) and appeal (*t*(115) = 3.61, *p* < 0.001) decreased after the training. Contrarily, divergence attitudes (toward acceptance) significantly increased (*t*(115) = − 19.19, *p* < 0.001) after the training. Since current EBPs lack guidance on accommodations for individuals with IDD and mental illness, these results support diverging attitudes from the strict fidelity of those models. When EBP models do not exist for a particular population, a caregiver must accommodate existing models which requires some level of divergence. Table 5 illustrates correlations between pre-training EBPAS scores and post-training EBP knowledge, and Table 6 shows post-training EBPAS scores and post-training EBP knowledge. Table 7 provides a summary of t test analyses between pre-EBPAS and post-EBPAS results.

**Table 5:** Correlations with pre-training evidence-based attitudes and post-training knowledge

	Knowledge	EBPAS	Requirement	Appeal	Openness	Divergence
Knowledge	1.000					
EBPAS	0.135	1.000				
Requirement	0.185	0.684**	1.000			
Appeal	0.064	0.786**	0.375**	1.000		
Openness	− 0.011	0.676**	0.156*	0.568**	1.000	
Divergence	− 0.075	− 0.537**	− 0.224**	− 0.182*	− 0.108	1.000

*EBPAS* Evidence-Based Practice Attitudes Scale total score, Requirement, Appeal, Openness, and Divergence are all subscales for the EBPAS

*p < 0.05; **p < 0.01

**Table 6:** Correlations with post-training evidence-based attitudes and post-training knowledge

	Knowledge	EBPAS	Requirement	Appeal	Openness	Divergence
Knowledge	1.000					
EBPAS	0.076	1.000				
Requirement	0.033	0.754**	1.000			
Appeal	0.063	0.856**	0.620**	1.000		
Openness	0.076	0.696**	0.257**	0.618**	1.000	
Divergence	− 0.029	− 0.208*	− 0.142	− 0.104	− 0.056	1.000

*EBPAS* Evidence-Based Practice Attitudes Scale total score; Requirement, Appeal, Openness, and Divergence are all subscales for the EBPAS

*p < 0.05; **p < 0.01

**Table 7:** Summary of pre-EBPAS and post-EBPAS scores

Scale/Subscale	Pre-Training		Post-Training		*t*	*p*
	*M*	SD	*M*	SD		
Total Score	45.41	7.29	35.26	8.20	15.26	0.001*
Requirements	9.16	2.95	8.02	3.96	3.34	0.001*
Appeal	12.43	2.64	11.48	3.09	3.61	0.001*
Openness	11.47	2.52	11.18	2.89	1.53	0.130
Divergence	3.68	2.24	11.43	2.78	− 19.19	0.001*

*N* = 116

*p < 0.01

## Discussion

The findings of this study indicate that the participants’ knowledge of evidenced-based strategies for working with individuals with co-occurring IDD and mental health conditions was acquired in a one-day training regardless of the participant’s amount of education. The post-test knowledge of the participants improved across the board, thus demonstrating that the training was effective at learning new information about this challenging area of mental health practice. Pre-test analysis indicates that participants with master’s degrees had more knowledge prior to the training than participants with less education, but the knowledge differences between the two groups disappeared upon post-test analysis. The similarities in post-test knowledge for participants with master’s degrees and those with less education is an important finding because it illustrates that educational degree is not necessarily an essential factor in knowledge acquisition for learning the adaptation of evidence-based strategies for this population. Additionally, this study concludes that the divergent attitudes for all participants increased. Given that there is no manualized, curriculum-based evidence-based model for this population, diverging attitudes toward traditional EBP models is viewed as a positive finding.

The applied research community and funding bodies place significant value on EBPs. However, individuals in need of services do not always fit a particular EBP model. Providers in complex community settings that extend far beyond hospital-based or general outpatient mental health settings (e.g., prisons, schools, and home-based environments) work with individuals for whom there is no established EBP and must have quick access to knowledge about ways to adapt their interventions. Although our participants maintained positive attitudes toward EBPs after the training, the increase in diverging attitudes illustrates that accommodations are not only necessary but welcomed among those caring for this population. When EBPs do not exist, a higher degree of deviation from a model is expected and supported in our results.

### Limitations and Future Research

The present study explored alternative training models and potential solutions for the workforce crisis. This research demonstrates that it is possible for all staff regardless of degree to obtain knowledge and alter attitudes in a relatively inexpensive and time-efficient manner. Research to determine if knowledge translates into practice is the next step to determining the effectiveness of task shifting. The study included data from only one training with participants in Nebraska, USA, which is a very serious limitation. Future training designs should include additional data collection on each of the areas of training in an effort to determine which areas carried the most potency in the package of training areas and include client outcome data. Furthermore, the convenience sample may result in data that are not representative of the entire population of direct staff working with individuals with IDD.

Consistent with this notion, as models become more sophisticated, larger sample sizes are required to statistically examine multiple group differences and maintain moderate effect sizes. Apparent in the second model exploring the effects of level of education, as covariates were added to the model, the model became marginally significant. It is likely that the covariates of age and gender influenced the results. However, the sample size of the bachelor’s level group included only 19 participants. Future research should explore these factors with a larger, more diverse sample size to disentangle this relationship and replicate the current findings. Integrating a control group would also strengthen the study.

In this era of workforce shortages, regardless of reason, task-shifting strategies have become more accepted practices across all human service disciplines (Rotheram-Fuller et al., 2017). However, it is yet to be determined if task shifting will be fully embraced by hierarchical systems designed to organize the workforce based on degree completion. Future research should examine the role of education but also staff background when adopting deviations to EBP models for specific population needs. While formal education explains some differences in core knowledge variation, it may also influence knowledge acquisition of trained materials and implementation of EBP adaptations. In addition, further exploration of informal education and experience should also be examined to determine their impact on training protocols. Lived experiences with family members, relatives, or friends with an intellectual disability, past volunteering experiences, and prior work in parallel fields may prove useful when educating and implementing EBP adaptations in the field. For instance, studies have shown that paraprofessionals learn and effectively implement EBP strategies when working in classrooms with students with Autism (Sam et al., 2022). Individuals with such backgrounds may possess a higher knowledge of EBPs and implement modifications more effectively or require less guidance given their prior experience. All of these factors offer a more comprehensive understanding of staffing factors that influence implementation of adaptive EBPs models.

The ability to adopt a new paradigm that removes degree completion barriers by the business sector, academia, and the individuals that comprise the workforce will likely be challenged. Moving toward a service system model that supports the adoption of matching task to talent and training to specific evidence-based strategies with clinical oversight, rather than typical hierarchical structures may further expand efforts of workforce inclusivity. The authors would like to point out that the project described in this manuscript outlines educating the workforce on core competencies, not advanced level skills reflective of what is required by individuals regardless of educational achievement.

Further limitations include the use of survey methods. Although a commonly accepted technique in the field, self-administered surveys only collect information at one point in time and are subject to response biases. Although the researchers attempted to maximize participation by offering incentives, these techniques do not ensure response accuracy and still risk socially desirable response patterns.

## Appendix

### Training Knowledge Competency Exam

1. Child management style is intended to
  - Describe normal variations in parental management
  - Describe deviant strategies associated with prolonged neglect or acute/chronic abuse
  - Connect specific caretaking strategies with specific child behaviors
  - Prescribe the best caregiving strategy to be used with all children
2. What is the primary role of adults who are responsible for children and adolescents?
  - Mentor and mimic appropriate behavior
  - Lead by example to show children appropriate decision-making into their adult lives
  - Influence, teach, and control the child’s behavior until the child can learn to control themselves
  - Influence and support a child’s natural exploration of the physical world.
3. Youth management styles primarily build off which two characteristics?
  - Nurturing and discipline
  - Responsiveness and demandingness
  - Warmth and discipline
  - Support and demand
4. In extreme cases, which parenting style more likely encompasses both rejecting–neglecting and neglectful management practices?
  - Indulgent
  - Authoritarian
  - Authoritative
  - Uninvolved
5. When comparing authoritative and authoritarian management styles, which style tends to offer more explanation and lower psychological control?
  - Authoritative
  - Authoritarian
6. When compared to promising practices, evidence-based practices (EBP) include additional requirements, such as
  - Multiple-site replications
  - Research published in peer review journals
  - At least one year of sustained effects
  - Only a and b
  - Only a and c
  - a, b, and c
7. Barriers to EBP implementation include as follows:
  - Not enough time
  - Not enough resources
  - Lack of training
  - All of the above
8. Which of the following interventions have not been “discredited?”
  - holding therapy
  - Dare drug abuse program
  - Systematic de-sensitization
  - Re-parenting therapies
9. Which of the following steps are not part of the EBP process of evaluating the evidence?
  - Formulate a well-structured answerable questions to address a practice need
  - Search the best available evidence to answer the question
  - Critically assess and evaluate the evidence for its validity, impact, and applicability to the situation
  - Integrate the evidence with clinical expertise and judgment and client wishes, values, and circumstances
  - Disseminate the evidence through peer reviewed journals
10. True or False: Adults with intellectual/development disabilities are three times more likely to suffer from mental illness compared to the general population.
11. What percentage of individuals with intellectual/developmental disability suffer from two or more diagnosed conditions?
  - 45%
  - 55%
  - 65%
  - 75%
12. Which of the following barriers have not been identified for individuals with dual diagnosis (comorbidity of at least 2 diagnosed conditions)?
  - Service delivery is fragmented
  - Funding and service eligibility
  - Failure to recognize population characteristics
  - No barriers
13. Barriers to diagnosis and assessment include all of the following except:
  - Communication deficits
  - Too much information
  - Lack of expertise
  - Atypical presentation of disorder or comorbid conditions
14. A 25-year-old male with schizophrenia and intellectual disability engage in outpatient mental health therapy to reduce aggression toward his caretakers. The therapist schedules 30-min sessions twice per week and sends home pictures with his caregivers to utilize during crises. The therapist is using __________________ to aid with treatment of the individual?
  - Therapeutic interventions
  - Therapy adaptations
  - EBP interventions
  - DD interventions
15. Why is more time needed to treat individuals with comorbid cognitive limitations?
  - Greater team collaboration and meetings
  - Data collection
  - Comprehensive evaluation
  - Identifying the best evidence in providing care and developing therapeutic adaptations
  - All of the above
16. Engagement requires all of the following except
  - Unconditional care and support
  - Activities that promote frequent interaction
  - Creating a judgment neutral environment
  - Generating reciprocal and mutually agreed upon expectations
17. Which of the following issues is the largest barrier to community living, relationship, employment, and staff retention when working with this population?
  - Challenging behavior
  - Comorbidity
  - Complex medical treatment
  - Difficult team or family members
18. A Functional Behavioral Assessment (FBA) differs from a comprehensive evaluation in the following way?
  - It provides background information of the individuals and his/her behaviors
  - It includes information from a team of clinicians and team members
  - It focuses on the why, how, where, when and what of the individuals’ behavior
  - It offers a broader evaluation of the individual
19. When completing an FBA, which step involves team members working to pull together information from the individual’s records, interviews, and questionnaires?
  - Step 1: Define the inappropriate behavior
  - Step 2: Collect, compare, and analyze information
  - Step 3: Hypothesize reasons for the behavior
  - Step 4: Develop a plan
20. True or False: Setting events are known as immediate or fast triggers, whereas antecedents are known as slow triggers.
21. Which of the following could be considered antecedents?
  - Pain
  - Poor sleep
  - Redirection
  - New setting
22. The process or over-attributing an individual’s symptoms to a particular condition, resulting in key comorbid conditions left undiagnosed and untreated refers to
  - Diagnostic overshadowing
  - Baseline exaggeration
  - Psychosocial masking
  - Cognitive disintegration
23. Due to a lack of cognitive reserve, some individuals with ID/DD may dramatically decompensate under stress resulting in difficulty communicating feelings of anxiety or distress and ultimately misdiagnosis, which is referred to as:
  - Diagnostic overshadowing
  - Intellectual distortion
  - Psychosocial masking
  - Cognitive disintegration
